# La fracture péri-prothétique de la hanche type C de Vancouver, ce qu'il ne faut pas faire

**DOI:** 10.11604/pamj.2016.23.5.8611

**Published:** 2016-01-17

**Authors:** Mohamed Amine Karabila, Ahmed Bardouni

**Affiliations:** 1 Service de Chirurgie Orthopédique et de Traumatologie, CHU Ibn Sina, Rabat, Maroc

**Keywords:** Fracture, prothèse, débricolage, Fracture, prosthesis, disassembly

## Image en médecine

Nous rapportons le cas d'une patiente âgée de 103 ans présentant une fracture récidivante de fémur droit sur prothèse de hanche avec débricolage de l'ancien matériel par défaillance de l'ostéosynthèse (A,B,C), puis reprise par une plaque verrouillée longue et cimentation des fragments osseux fragiles (D). A six mois de recul, la fracture a consolidé sans déplacement et sans signe de descellement (E). L'analyse de ce cas montre qu'il ne faut pas se lancer dans une ostéosynthèse sans une programmation minutieuse et ne doit pas être réalisée dans l'urgence. Le souci d'une planification soigneuse, avec des implants spécifiques, respectant les principes fondamentaux de l'ostéosynthèse (6 à 8 corticales de part et d'autre de la fracture et un bon chevauchement des 2 implants), doit devenir une priorité afin d’éviter tout traitement minimaliste conduisant à un échec thérapeutique.

**Figure 1:**
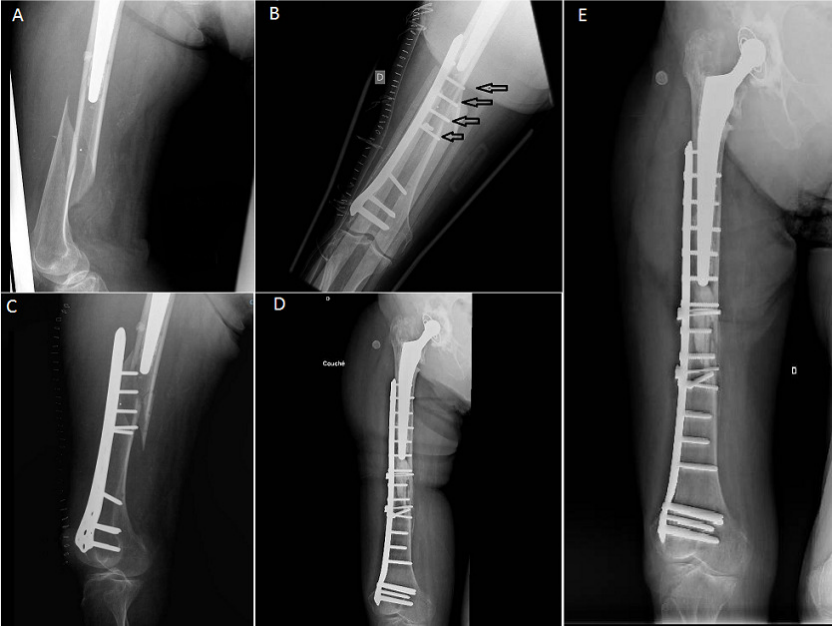
A) fracture spiroide type C de vancouver; B) ostéosynthèse initiale par plaque courte, seulement 4 vis bi-corticales; C) déplacement secondaire par manque de stabilité de l'ostéosynthèse; D) contrôle post-opératoire de la reprise par une plaque longue à tête verrouillée, cerclage et cimentation du foyer fracturaire; E) contrôle après 6 mois

